# Risk-adapted neck dissection in salivary gland carcinoma: integrating occult nodal metastasis patterns with contemporary guidelines

**DOI:** 10.3389/fonc.2026.1916521

**Published:** 2026-08-24

**Authors:** Felix Johnson, Lea Stecher, Nora-Maria Burian, Marcel Kloppenburg, Rubens Thölken, Benjamin Freytag, Patrick Schuler, Benedikt Hofauer

**Affiliations:** 1 Department of Otorhinolaryngology, Head and Neck Surgery, Heidelberg University Hospital, University of Heidelberg, Heidelberg, Germany; 2 University Hospital for Otorhinolaryngology, Medical University of Innsbruck, Innsbruck, Austria

**Keywords:** clinical guidelines, frozen section analysis, neck dissection, occult nodal metastasis, parotid gland carcinoma, salivary gland carcinoma, submandibular gland tumor

## Abstract

**Introduction:**

Salivary gland carcinomas (SGCs) are rare and histologically diverse malignancies of the head and neck. They present significant challenges for standardized treatment due to varied metastatic behavior and limited high-level evidence. The extent of neck dissection remains controversial, influenced by tumor grade, histological subtype, and anatomical origin. This review evaluates current evidence and guideline recommendations to define an evidence-based, risk-adapted approach to neck dissection in SGC.

**Methods:**

A comprehensive review of epidemiological data, retrospective cohort studies, meta-analyses, and major clinical guidelines was conducted. Particular attention was given to patterns of cervical metastasis, the impact of histological grading on management, complication rates, and diagnostic tools that guide surgical planning.

**Results:**

The incidence of occult nodal metastases (ONM) varies widely by tumor type and grade, ranging from 10% to over 60% in aggressive subtypes. Low-grade tumors with intraglandular spread often warrant selective neck dissection (levels I–III), while high-grade or T3–T4 tumors benefit from more comprehensive neck dissection (levels I–V) due to increased risk of skip metastases. Preoperative imaging, Core Needle Biopsy (CNB), and intraoperative frozen sections are essential to accurately assess risk and avoid the morbidity associated with delayed or revision surgery.

**Conclusion:**

A one-stage, risk-adapted neck dissection strategy tailored to tumor biology and diagnostic findings improves oncological outcomes and minimizes complications. While low-risk cases may be managed with selective or limited neck dissection, high-risk SGCs require comprehensive management to achieve regional control. Standardized protocols based on robust prospective data remain an unmet need.

## Introduction

Epidemiological studies have shown that salivary gland carcinomas (SGCs) account for an estimated 6-8% of head and neck cancers, with an overall incidence of 1.1 salivary gland tumors per 100,000 inhabitants (1–3). Up to 20% of salivary gland tumors (SGTs) are found to be malignant on histological examination. Tumors of the parotid gland are the most common neoplasms of the salivary glands, of which about 20% are malignant, whereas tumors of the submandibular gland are malignant in 43% of cases (4, 5). Only about one third of malignant tumors show typical signs of malignancy from the outset, such as facial nerve paralysis, pain or infiltration of the skin (6). In addition, it is often not possible to clearly differentiate between benign and malignant tumors in imaging, which can sometimes result in the surprising diagnosis of a malignant tumor after surgical removal of the tumor (7–9).

SGCs currently encompass a group of diverse tumors with 21 histological subtypes (5, 10, 11). The wide range of subtypes combined with the different locations where the primary tumors can occur, reflect the difficulty in defining standardized guidelines for treatment options and surgical concepts, especially in terms of the extent of a neck dissection.

Determining the optimal extent of neck dissection in cases of salivary gland carcinoma therefore remains controversial. Different studies and guidelines advocate varying extents of neck dissection, generally recommending selective dissection in clinically node-negative patients while reserving more comprehensive dissections, including level V, primarily for clinically node-positive disease or selected high-risk situations. This decision is influenced by factors such as tumor histological subtype, clinical stage, and lymphatic drainage patterns, as well as by the risk of occult nodal metastases and potential operative morbidity. Different clinics recommend or perform different neck dissections often based on institutional experience and surgeon preference, leading to variability in management strategies, which underlines the lack of an international standardized guideline and clear algorithms for treatment regarding the surgical extent.

Current international guidelines demonstrate partially divergent recommendations regarding the indication and anatomical extent of neck dissection in salivary gland carcinoma. While there is broad consensus that clinically node-positive (cN+) disease warrants comprehensive neck dissection, meaningful variability emerges in the management of the clinically node-negative (cN0) neck. Differences primarily concern the threshold for elective treatment and the extent of levels removed. The German S3 guideline adopts a comparatively conservative approach, permitting observation in selected cN0 cases and emphasizing selective dissection (12).

In contrast, the current NCCN Head and Neck Cancer Guidelines (Version 1.2026) and ASCO more consistently recommend elective neck dissection in high-grade or T3–T4 tumors, occasionally implying broader anatomical extension in advanced disease, whereas therapeutic neck dissection remains the standard for clinically node-positive disease (13). ESMO–EURACAN proposes a biologically risk-adapted strategy, linking elective treatment to estimated risk of occult nodal metastasis.

Importantly, however, most guidelines base their recommendations predominantly on surrogate markers such as histologic grade and primary tumor stage, without providing detailed, histology-specific estimates of occult nodal metastasis (ONM) rates across the wide spectrum of salivary gland carcinoma subtypes. As a result, surgeons are often required to interpret heterogeneous retrospective data independently when deciding whether elective neck dissection is indicated and which cervical levels should be included. The present review therefore seeks to consolidate and contextualize the available evidence on tumor-specific ONM patterns, offering a concise, biologically grounded framework to support risk-adapted and anatomically rational neck management beyond stage- and grade-based guidance alone.

## Impact of histological grading on management

Salivary gland cancers are a rare group of heterogenous tumor entities categorized by the WHO into 21 different types. While the WHO defines the histological categories, the stratification into distinct clinical entities based on grading reflects a clinically driven approximation—guided by how tumor grade influences patient management (**Table 1**). Histological grading of salivary gland carcinomas emerged from the clinical observation that tumors of the same histopathological type exhibited markedly different biological behaviors. This discrepancy prompted retrospective pathological analyses, which revealed consistent correlations between certain microscopic features—such as increased mitotic activity, differentiation, invasion, nuclear pleomorphism, necrosis, and architectural patterns—and clinical outcomes. In response, grading systems were developed to stratify tumors based on these histological criteria, enabling more accurate prognostication and informing therapeutic decisions. Over time, these systems have been refined and validated, solidifying grading as a critical component in the clinical management of several salivary gland carcinoma subtypes. Not all SGCs have clinically relevant grading, and grading classifications may be variable.

**Table 1:** Histologic subtypes and grading relevance in SGC (11).

Histologic subtype	Grading system	Clinical risk stratification
Mucoepidermoid carcinoma	Low/Intermediate/High	Strong prognostic impact
Acinic cell carcinoma	Low/High	Prognostically relevant
Adenoid cystic carcinoma	Tubular/Cribriform/Solid	Limited nodal impact
Carcinoma ex pleomorphic adenoma	Invasion depth–based	High if invasive
Salivary duct carcinoma	Intrinsically high-grade	High risk
Small cell neuroendocrine carcinoma	High-grade	High risk
Large cell neuroendocrine carcinoma	High-grade	High risk
Primary salivary SCC	High-grade	High risk
Secretory carcinoma	Usually low-grade	Generally favorable
Basal cell adenocarcinoma	Low/High	Variable
Epithelial-myoepithelial carcinoma	Variable	Intermediate
Myoepithelial carcinoma	Variable	Intermediate–high
Polymorphous adenocarcinoma	Limited grading value	Low risk
Clear cell carcinoma	Not routinely graded	Low risk
Oncocytic carcinoma	Not routinely graded	Variable
Lymphoepithelial carcinoma	Not routinely graded	Often aggressive
Adenocarcinoma NOS	Variable	Heterogeneous
Sebaceous carcinoma	Rare	Variable
Cribriform adenocarcinoma	Rare	Variable
Hyalinizing clear cell carcinoma	Rare	Low risk
Sialoblastoma	Pediatric entity	Borderline

Recommendations often vary by histological grading (e.g., low-, intermediate-, or high-grade), as grading significantly impacts clinical behavior and treatment decisions. For example, low-grade mucoepidermoid or acinic cell carcinomas may follow an indolent course requiring limited intervention, whereas their high-grade counterparts can behave aggressively and necessitate more extensive therapy. When grading-related clinical behavior is considered, the number of functionally distinct salivary gland carcinoma (SGC) entities exceeds the 21 histological subtypes defined by the WHO and likely approaches 35–40 (see **Table 1**).

As a result, the rarity of distinct histological subtypes, variability in grading, and the diverse primary tumor locations—including the parotid, submandibular, sublingual, and minor salivary glands—make data pooling challenging. For some of the more common tumor entities—such as mucoepidermoid carcinoma, adenocarcinoma, epithelial-myoepithelial carcinoma, and oncocytic carcinoma—sufficient study cohorts (>100 cases) exist to support confident recommendations regarding both the indication and extent of neck dissection, even across different histological grades (14–17).

A retrospective study showed no strong association between grading classifications for adenoid cystic carcinoma and risk of cervical lymph node metastasis (18). However, data on cervical lymph node metastasis rates are largely retrospective and limited by small cohort sizes. Many studies are outdated, meaning that the ability to determine positive clinical lymph node status (cN+) is poor as compared to today due to improved imaging methods. Furthermore, the changing landscape of the classification of SGCs makes comparing older studies with new publications difficult.

**Figure 1:**
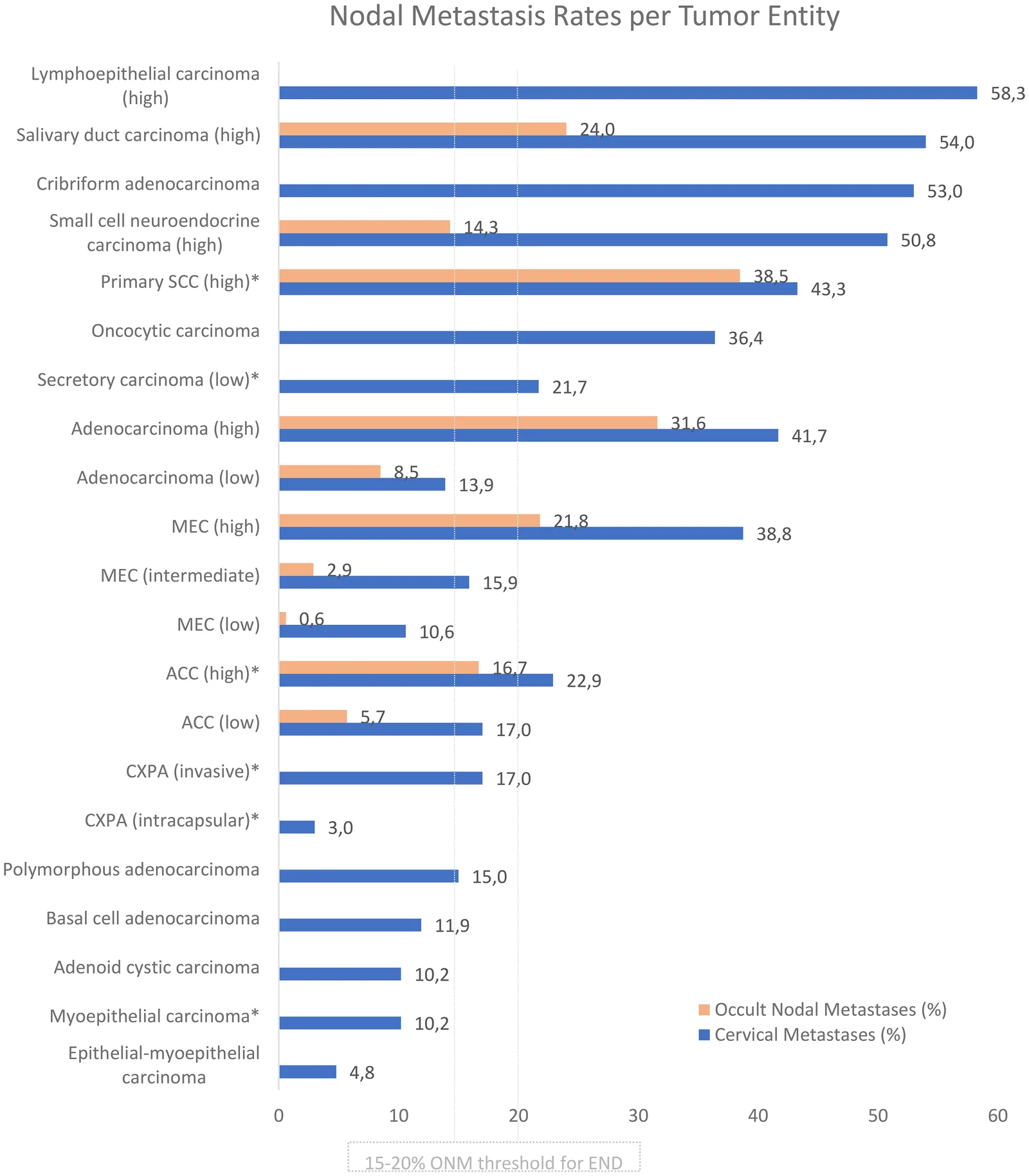
Nodal metastasis rates per tumor entity. Data obtained from (14, 19, 20): (15–18, 21–34) SCC, Squamous cell carcinoma; MEC; Mucoepidermoid carcinoma; ACC, Acinic cell carcinoma; CXPA, Carcinoma ex pleomorphic adenoma; END, Elective Neck Dissection; ONM, Occult Nodal Metastasis. *Data limited (under 50 cases).

A summary of the available data describing nodal metastasis rates including clinically apparent metastasis and occult metastasis is seen in **Figure 1**.

## Lymphatic drainage and metastatic patterns

Salivary gland tumors, whether arising in the parotid or submandibular glands, demonstrate distinct lymphatic drainage. Parotid carcinomas most frequently metastasize to levels II and III, although high-grade tumors or those with extracapsular spread may also involve level I and occasionally extend to levels IV or V. In contrast, submandibular gland carcinomas typically metastasize to levels I and II, with some evidence indicating occasional involvement of level III in aggressive disease. These drainage patterns support tailoring the extent of neck dissection to the individual risk profile.

## Impact of primary salivary gland site

**Figure 2:**
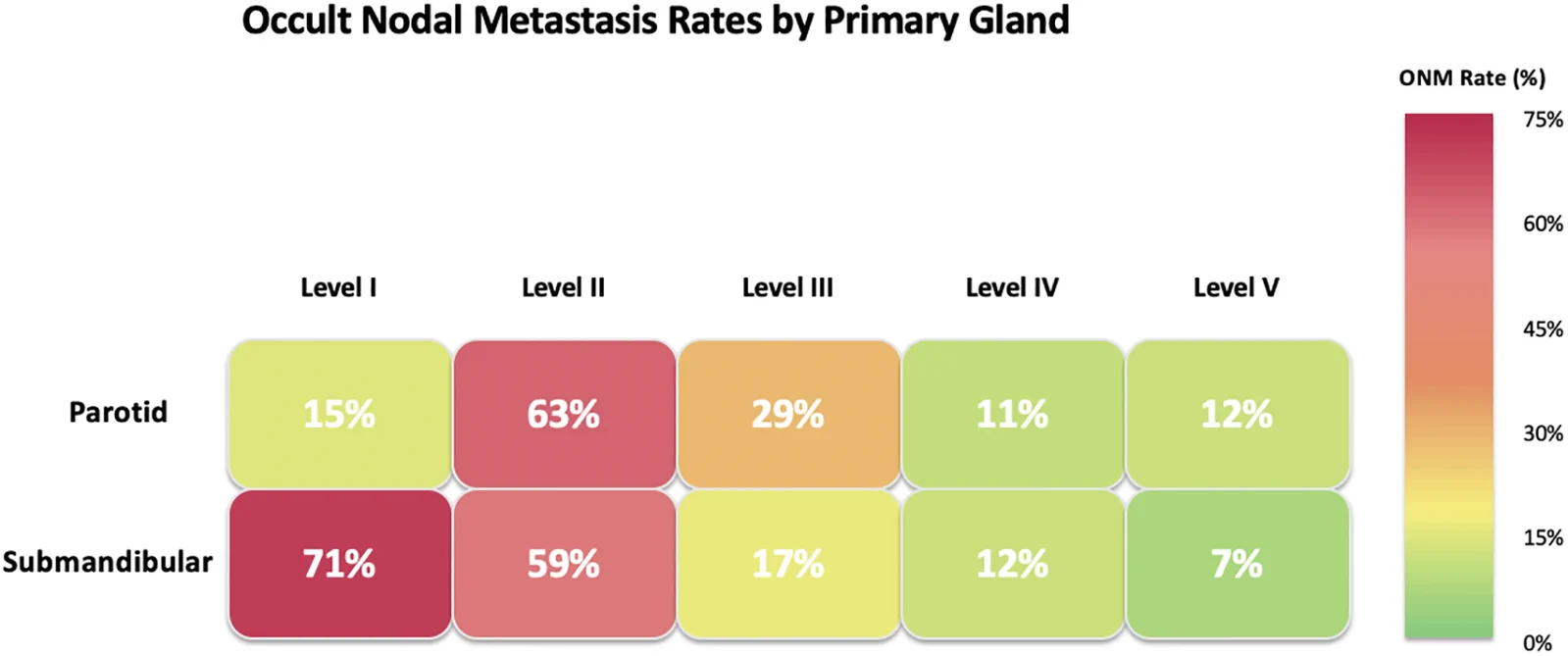
Heat-map with data illustrating ONM rates stratified according to primary tumor site (adapted from Yan et al., Head Neck. 2022 (35).

The likelihood and anatomical distribution of lymph node metastases are closely related to the primary tumor site (see **Figure 2**). Tumors of the minor salivary glands exhibit more variable patterns of lymphatic drainage depending on their anatomical location, often requiring a tailored approach to neck management.

Prognosis is also strongly site-dependent. Parotid gland tumors are generally associated with more favorable outcomes, largely due to earlier detection and the predominance of less aggressive histologic subtypes. In contrast, tumors arising in the submandibular, sublingual, and minor salivary glands tend to present at a more advanced stage and are linked with a higher risk of adverse outcomes (36).

Histologic subtype further influences both tumor behavior and site distribution. For example, mucoepidermoid carcinoma most commonly arises in the parotid gland, while adenoid cystic carcinoma frequently originates in the minor salivary glands. These histologic-site correlations have prognostic significance, particularly given the variable risks of perineural invasion and distant metastasis among subtypes. Together, these factors are essential in guiding decisions regarding the extent of neck dissection and overall treatment planning.

## International and German guidelines

The guidelines consistently emphasize the necessity and recommendation of a preoperative fine-needle biopsy as a standard diagnostic procedure (see **Table 2**). While NCCN recommends FNA as initial tissue diagnosis, both S3 and ASCO allow either FNA or CNB. Given the increasing relevance of histologic subtyping and biomarker testing in salivary gland malignancies, we favor primary ultrasound-guided CNB in cases with imaging features suggestive of malignancy.

**Table 2:** Recommended diagnostic procedures.

Diagnostic workup in major salivary gland tumors—guideline comparison								
Guideline	Clinical exam	US	FNA	CNB	MRI	CT neck	CT chest	PET/CT
ESMO 2022	✓	✓	✓	Optional	✓	Advanced disease	T3–4 / AdCC	cN+ / high-grade
S3 2024	✓	✓*	✓	Optional	✓	Bone invasion	—	Adjunct role
NCCN 2023	✓	Optional	✓	Optional	✓	Optional	Optional	Advanced disease
ASCO 2021	✓	✓	✓	Optional†	✓‡	Bone invasion	High-risk	High-grade

Exam, clinical ENT examination; US, ultrasound; FNA, fine needle aspiration cytology; CNB, core needle biopsy; opt, optional; adv, advanced disease; adj, adjunct role; HG, high-grade tumor; HR, high-risk disease; AdCC, adenoid cystic carcinoma; bone, suspected bone invasion; cN+, clinically node-positive; T3–4, tumor stage 3–4; *recommended initial imaging modality, †recommended when FNA is nondiagnostic, ^‡^recommended when malignancy suspected or concern for nerve or skull base involvement.

A common recommendation amongst current guidelines, including the recent S3-German guideline, in patients with cN0 lymph node status, is to recommend an elective neck dissection in cases of high-risk occult lymph node metastasis.

The German S3 guideline “Diagnosis and Therapy of Salivary Gland Tumors” advises a risk-adapted approach. In cases of either high-grade histology, a T3-T4 status, or ECE, an elective ipsilateral cervical neck dissection should be performed even in cases without suspicion of local lymph node metastasis.

In cN0 cases, for low-grade tumors a watch-and-wait strategy may be sufficient; however, high-grade tumors and tumors with advanced T-stage warrant a selective neck dissection (levels II–IV for parotid tumors and I-III for submandibular tumors). In cases of pretherapeutic positive cervical lymph nodes a neck dissection of levels I-V be performed.

The ESMO–EURACAN Clinical Practice Guideline (2022) recommends that therapeutic neck dissection be considered in patients with clinically positive nodes (cN+) or those with high-risk features even in a cN0 neck, basing the dissection on the anticipated drainage basin than a uniform strategy.

In addition to evolving guideline recommendations, the recently introduced 9^th^ edition of the AJCC/UICC TNM classification further emphasizes the prognostic significance of cervical lymph node involvement by defining the N category not solely based on lymph node size, but rather on the number of metastatic lymph nodes and extranodal spread. This modification reflects accumulating evidence that nodal burden is a stronger prognostic factor than the size of individual metastatic lymph nodes. Although this revision primarily affects pathological staging rather than the indication for elective neck dissection, it further emphasizes the importance of accurate cervical nodal assessment within contemporary risk-adapted management strategies.

**Table 3** presents a comparative analysis of the recommended diagnostic pathways across different guidelines.

**Table 3:** International and german guidelines recommendations.

Neck management in major salivary gland carcinoma—guideline comparison					
Guideline	cN+ disease any T, any grade	cN0 – parotid T1–T2, low/intermediate grade	cN0 – parotidT3–T4 and/or high grade	cN0 – submandibular low grade, intraglandular	cN0 – submandibular high grade or extraglandular
ESMO–EURACAN 2022	Therapeutic ND (levels per disease extent)	Selective ND II–III (consider II–IV); sentinel possible	END II–IV (Ib/V if indicated)	END I–III recommended	END I–III (± IV by histology)
German S3 Guideline 2024	Therapeutic ND (“comprehensive” recommended)	Observation acceptable in low-risk constellation	END II–IV	END I–III	END I–III
NCCN 2023	Therapeutic ND (extent per staging)	No routine END (low-risk)	END recommended (levels not specified)	No routine END (low-risk)	END recommended (levels not specified)
ASCO 2021	Therapeutic ND (levels per disease extent)	Observation reasonable; END individualized	Offer END (if performed: II–IV)	Observation or END I–III	Offer END (levels site-dependent)

## Evaluating the role of elective neck dissection in clinically N0 neck

**Figure 3:**
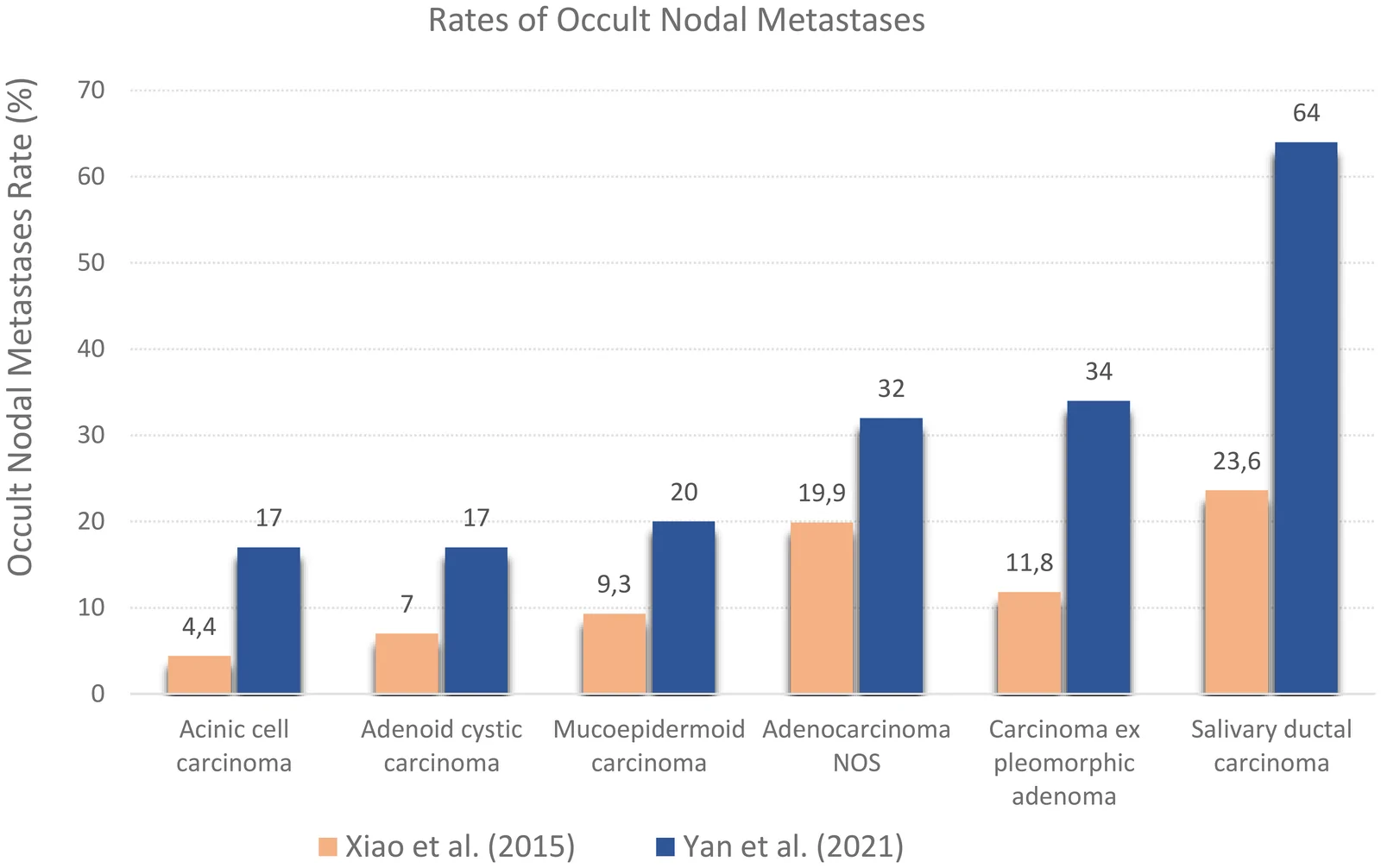
Rate of occult nodal metastases regardless of grading.

Large epidemiological studies and metanalyses of thousands of patients have shown that cervical metastases occur in 24.4% of cases, and occult nodal metastases in 10.2% of cases (15). Patients with clinically node-negative (N0) status and low-grade disease have significantly better 5-year overall survival compared to those with nodal metastases (N+) or high-grade tumors (79% vs. 40% and 88% vs. 69%, respectively; both P <.001) (15). However, rates of lymph node metastases and ONM are highly dependent upon the tumor entity, with rates of ONM as high as 64% for the salivary duct carcinoma (see **Figure 3**). Patients with T3/T4 tumors have a reported 2.3 times higher risk of ONM as compared to T1/T2 (15, 35).

## Evidence supporting limited versus comprehensive neck dissection

Retrospective cohort studies have demonstrated that in many parotid and submandibular carcinomas, the majority of metastatic nodes are found in levels II and III. For instance, Nobis et al. reported that a limited neck dissection (levels I–III) adequately addressed occult metastases in low-risk cases, whereas higher-grade tumors exhibited skip metastases into levels IV and V—supporting a more extensive dissection in such scenarios. Similarly, Vander Poorten et al. found that submandibular gland carcinomas predominantly metastasize to level I, with occasional involvement of levels II and III in high-risk cases.

A meta-analysis conducted by Flora Yan et al. systematically reviewed retrospective studies on elective neck dissection in salivary gland carcinomas published between 1973 and 2020. A total of 51 studies were included, encompassing 11,698 patients with a mean age of 57.1 years. The analysis indicates that there is still no definitive consensus on the indications for elective neck dissection in a cN0 neck, particularly regarding the extent of the procedure and the circumstances under which observation may be deemed sufficient. The study identified that metastases in the cervical lymph nodes predominantly occur at levels I, II, and III, with incidence rates of 30%, 65%, and 36%, respectively. The metastatic pattern in level I varies depending on the primary tumor location within the parotid or submandibular gland. Metastatic involvement at level I is significantly lower in parotid tumors (15%) compared to submandibular carcinomas (71%). The results suggest that at least levels II and III should be routinely assessed in elective neck dissection for malignant salivary gland tumors. For submandibular gland carcinomas, additional resection of level I is appropriate.

## Radical neck dissection: historical data and metastatic patterns

Earlier studies conducted during the era when elective radical neck dissections were routinely performed have provided valuable insight into the metastatic patterns of salivary gland carcinomas. These comprehensive dissections revealed that while lymph node metastases predominantly involved levels I–III, certain high-grade tumors also demonstrated involvement of levels IV and V, thereby supporting the role of extended dissections in select high-risk cases. Although these data remain clinically informative in identifying patients who may benefit from broader neck management, reported rates of occult nodal metastasis (ONM) from that period are not directly comparable to contemporary figures, given substantial advancements in preoperative imaging and diagnostic accuracy (37).

## Oncological rationale and complication rates

Given the surgical burden, understanding potential complications is critical. Oncologically, the goal is to remove nodal basins at high risk of harboring metastases while minimizing operative morbidity. A meta-analysis by van Herpen et al. demonstrated that, in cN0 necks, occult metastases were most commonly located in levels II and III, thereby supporting limited dissection for low- to intermediate-risk tumors. In contrast, high-risk patients—with high-grade histologies such as salivary duct carcinoma or carcinoma ex pleomorphic adenoma—benefit from an extended neck dissection (levels I–V) to remove possible skip metastases and improve regional control.

Neck dissection, however, is not without risk. When the procedure is postponed to a separate, second-stage operation, scar tissue formation complicates dissection and increases the risk of injury to key structures. For example, accessory nerve injury during revision neck dissection has been associated with shoulder dysfunction in 10–20% of cases, while the marginal mandibular nerve is injured in 1–5% of cases. Other complications, including chyle leak (1–3%), hematoma, and infection (each around 2–5%), tend to be more frequent in the reoperative setting due to distorted anatomy and fibrosis.

## Preoperative and intraoperative evaluation to optimize neck dissection

A major challenge in managing salivary gland carcinoma is that definitive histological grade and margin status are often determined only after tumor resection. This leaves surgeons without crucial information to base the decision for elective neck dissection at the time of primary surgery. If neck dissection is deferred to a later stage, increased operative complications from tissue scarring and distorted anatomy are encountered.

Preoperative evaluation methods such as fine needle aspiration (FNA), core needle biopsy (CNB) and intraoperative frozen section analysis provide additional histopathological information. CNB can yield data regarding tumor grade and cell type, with sensitivity of 92% and high specificities approaching 100% in salivary gland lesions, although its accuracy may be limited by sampling error (38). Intraoperative frozen section analysis is recommended in unclear cases to determine malignity and additionally allows for immediate assessment of margins during the primary surgery, thereby guiding the extent of resection and neck dissection. The intraoperative utilization of frozen section analysis is recommended for cases with preoperatively unclear histological findings of submandibular gland tumors. Intraoperative section analysis is particularly effective in distinguishing benign masses and can help reduce the need for additional surgical interventions (39).

Advanced preoperative imaging with CT or MRI is equally essential. These modalities assess tumor size and detect extracapsular spread—strong predictors for occult nodal metastasis that can influence the decision to perform a more comprehensive neck dissection.

## Tailoring neck dissection based on adjuvant therapy responsiveness

Some salivary gland malignancies, including acinic cell carcinoma, are primarily treated surgically and show limited responsiveness to systemic therapies. While postoperative radiotherapy can improve local control in high-risk cases, its benefit in low-grade tumors remains less clear. This is likely related to their indolent growth patterns, low cellular turnover, and a clinical course characterized more by distant metastasis or perineural invasion than by locoregional recurrence. On the other hand, aggressive subtypes such as salivary duct carcinoma and high-grade mucoepidermoid carcinoma often derive measurable benefit from adjuvant radiotherapy, especially in achieving regional disease control (40). Given these differences in therapeutic responsiveness, the scope of neck management should be tailored accordingly. Tumors with a low likelihood of responding to adjuvant modalities may require a more definitive surgical clearance of cervical lymphatics during initial treatment. In contrast, when dealing with tumor entities known to respond well to adjuvant therapy, a conservative neck dissection may be justified in the absence of clinical nodal involvement, relying on adjuvant therapy to address any residual microscopic disease.

## Comparative analysis and future perspectives

The decision to perform limited (i.e. levels I–III) versus comprehensive neck dissection (levels I–V) must be individualized based on tumor characteristics, imaging findings, and intraoperative assessments. Although limited dissection may reduce operative time and morbidity, it risks incomplete clearance in high-risk tumors. Comprehensive dissection increases the likelihood of complete nodal clearance at the expense of higher complication rates.

Future randomized controlled trials are needed to definitively determine the balance between oncological benefits and morbidity risks associated with each dissection strategy. Meanwhile, multidisciplinary decision-making—integrating input from head and neck surgeons, radiologists, and pathologists—remains essential.

The extent of neck dissection in submandibular gland carcinomas is significantly influenced by the presence of extracapsular extension (ECE+) and histological grading. High-grade tumors and those demonstrating ECE+ have an increased likelihood of nodal metastasis beyond levels I–III, necessitating a more extensive dissection to improve regional control. Conversely, low- to intermediate-grade tumors without ECE+ may be adequately managed with a limited dissection, thereby reducing the risk of postoperative complications.

To optimize surgical planning and reduce surgical comorbidities, preoperative CNB and intraoperative frozen section analysis are critical. CNB provides early cytological insight with high specificity (41), helping to stratify patients based on their risk of nodal involvement; and when histology remains unclear the intraoperative frozen section analysis may help confirm malignity.

Preoperative radiologic imaging, including MRI and CT, can help determine the presence of ECE+ and inform the surgical approach. Furthermore, intraoperative assessment of extracapsular spread allows for real-time adjustment of the dissection extent. If ECE+ is observed during surgery, expanding the neck dissection to additional levels (e.g., IV and V) may be warranted to achieve optimal oncological outcomes.

Incorporating this multimodal strategy—integrating preoperative imaging, FNA, and frozen section analysis—facilitates a more tailored approach. This approach enhances operative results by ensuring that the extent of neck dissection is precisely matched to the tumor’s biological behavior, thereby balancing effective oncologic control with the minimization of surgical morbidity.

**Figure 4:**
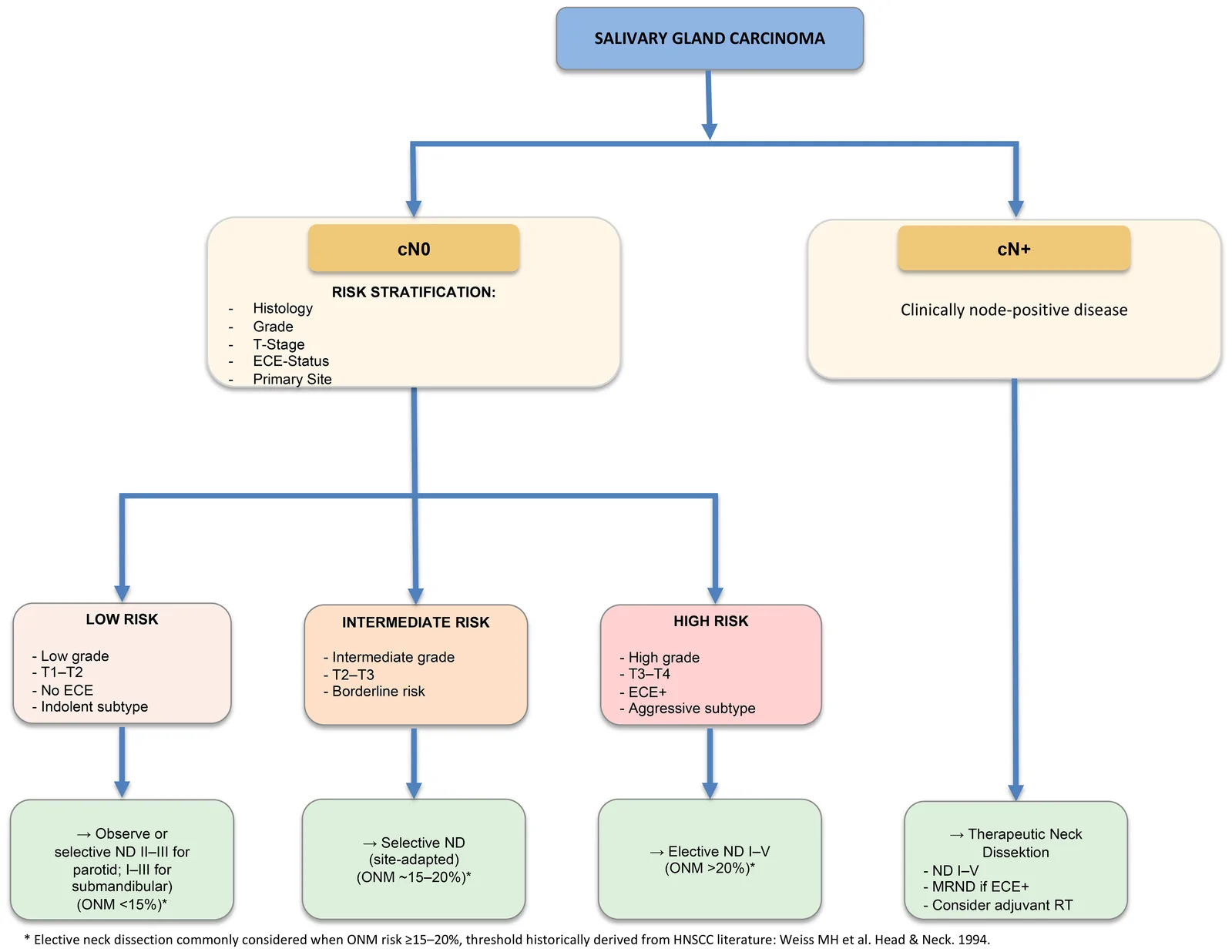
Risk-adapted algorithm for cervical lymph node management in salivary gland carcinoma.

In clinically node-negative (cN0) patients, risk stratification (see **Figure 4**) is based on histology, tumor grade, T-stage, extranodal extension (ECE) status, and primary tumor site. Patients are categorized into low-, intermediate-, and high-risk groups according to estimated occult nodal metastasis (ONM) risk. Elective neck dissection is considered when ONM risk exceeds 15–20%, with increasing extent of nodal levels addressed in higher-risk groups. In clinically node-positive (cN+) disease, therapeutic neck dissection is recommended, with modification based on ECE status and consideration of adjuvant radiotherapy.

## Conclusions

The literature supports a risk-adapted, one-stage approach to neck dissection in salivary gland carcinoma. In patients with low- to intermediate-risk tumors, limited neck dissection (levels I–III) may be oncologically sufficient while minimizing morbidity. In contrast, high-grade or clinically node-positive disease necessitates a comprehensive approach (levels I–V) to ensure complete clearance of metastatic nodes. Preoperative imaging, FNA, and intraoperative frozen section analysis are invaluable tools that provide critical information to guide surgical planning. This integrated approach not only improves oncological outcomes but also reduces the complication rates associated with reoperative neck dissection due to scar tissue formation.
